# Nitrotyrosine, Nitrated Lipoproteins, and Cardiovascular Dysfunction in Patients with Type 2 Diabetes: What Do We Know and What Remains to Be Explained?

**DOI:** 10.3390/antiox11050856

**Published:** 2022-04-27

**Authors:** Grzegorz K. Jakubiak, Grzegorz Cieślar, Agata Stanek

**Affiliations:** Department and Clinic of Internal Medicine, Angiology, and Physical Medicine, Faculty of Medical Sciences in Zabrze, Medical University of Silesia, Batorego 15 St., 41-902 Bytom, Poland; cieslar1@tlen.pl (G.C.); astanek@tlen.pl (A.S.)

**Keywords:** 3-nitrotyrosine, nitrated lipoproteins, cardiovascular disease, diabetes mellitus, oxidative stress, myeloperoxidase

## Abstract

Diabetes mellitus (DM) is a strong risk factor for the development of cardiovascular diseases (CVDs), which are the most important cause of morbidity and mortality in the population of patients living with DM. DM is associated with lipid metabolism disorders characterized by a decrease in the high-density lipoprotein blood concentration, an increase in the triglyceride blood concentration, and the presence of modified lipoproteins not routinely measured in clinical practice. Nitrated lipoproteins are produced by the nitration of the tyrosyl residues of apolipoproteins by myeloperoxidase. There is some evidence from the research conducted showing that nitrated lipoproteins may play a role in the development of cardiovascular dysfunction, but this issue requires further investigation. It was found that the nitration of HDL particles was associated with a decrease in caspase-3 and paraoxonase-1 activity, as well as a decrease in the activity of cholesterol transport via ABCA1, which reduces the protective effect of HDL particles on the cardiovascular system. Less information has been collected about the role of nitrated LDL particles. Thus far, much more information has been obtained on the relationship of nitrotyrosine expression with the presence of cardiovascular risk factors and the development of cardiovascular dysfunction. The purpose of this paper is to provide an extensive review of the literature and to present the most important information on the current state of knowledge on the association between nitrotyrosine and nitrated lipoproteins with dysfunction of the cardiovascular system, especially in patients living with DM. Moreover, directions for future research in this area were discussed.

## 1. Introduction

### 1.1. Cardiovascular Disease in Patients Living with Diabetes Mellitus

Diabetes mellitus (DM) and its complications are one of the most important problems in public health worldwide. The number of patients living with DM is systematically increasing, which generates significant costs for healthcare systems [1]. DM is well documented to be a strong risk factor for the development of cardiovascular diseases (CVDs). DM predisposes the development of microvascular and macrovascular complications. CVDs are the leading cause of morbidity and mortality in the population of patients living with DM [2].

Coronary heart disease (CHD), ischemic cerebrovascular disease (ICVD), and peripheral arterial disease (PAD) are the most important clinical manifestations of atherosclerotic CVD [3]. DM accelerates and modifies the course of atherosclerotic CVD. PAD in patients with DM is characterized by a predisposition to multilevel stenosis and occlusion, as well as arteries below the knee more often being affected [4]. DM is also a risk factor for restenosis in patients after percutaneous balloon angioplasty with or without stent implantation, which worsens the prognosis and may lead to the need for reintervention [5].

### 1.2. Lipid Disorders in the Course of Diabetes Mellitus

The parameters of the lipid profile routinely determined in clinical practice, such as the concentrations of total cholesterol (TC), low-density lipoprotein (LDL) cholesterol (LDL-C), high-density lipoprotein (HDL) cholesterol (HDL-C), and triglycerides (TG), provide only some information about the state of a patient’s lipid metabolism, because lipoproteins can undergo different modifications in vivo that alter their pro- or anti-atherogenic profile. The mentioned modifications can be of enzymatic or non-enzymatic character. Lipoproteins can be modified through oxidation, glycation, desialylation, carbamylation, nitration, and chlorination, among other processes [6,7,8].

Lipid metabolism has been shown to be disordered in patients living with DM. The typical patterns of dyslipidemia in T2DM include an increased TG level, decreased HDL-C level, and higher susceptibility to forming more atherogenic small dense low-density lipoproteins (sdLDLs) [9]. In patients with normal sensitivity to insulin, insulin inhibits the secretion of very low-density lipoproteins (VLDLs) from the liver [10]. Insulin resistance (IR) is associated with a decrease in the activity of lipoprotein lipase, leading to the ineffective clearance of VLDL cholesterol and an increase in its blood concentration. Moreover, an increased plasma level of VLDL leads to a situation in which the plasma cholesteryl ester transfer protein (CETP) exchanges the triglycerides in VLDL for cholesterol in HDL. This is associated with the formation of more atherogenic VLDL particles and less protective HDL particles [10,11]. IR is associated with the inhibition of lipogenesis and stimulation of lipolysis, leading to an increase in the blood concentration of free fatty acids (FFAs) [12]. An increased delivery of FFAs to peripheral tissue activates the production of triglyceride-rich lipoproteins [10]. Moreover, an increase in the level of FFAs leads to the exacerbation of IR [13].

In Table 1, the results of selected studies contributing to the understanding of the cellular and molecular mechanisms responsible for the pathogenesis of dyslipidemia in the course of IR are shown.

### 1.3. Modified Lipoproteins in Atherogenesis

Modified lipoproteins promote the development of the inflammatory process within the arterial wall, macrophage activation, and the synthesis and secretion of proinflammatory cytokines, chemokines, and enzymes. The following modified lipoproteins were shown to play a role in the pathogenesis of CVDs: vortexed LDL, acetylated LDL, electronegative LDL, enzymatically modified LDL, lysosomal acid lipase-modified LDL, phospholipase A2-modified LDL, sphingomyelinase-modified LDL, oxidized LDL, oxidized VLDL, carbamylated LDL, carbamylated HDL, and advanced glycation end-product-modified LDL [23]. Glycated lipoproteins, which may be of particular interest for patients with DM, are also characterized by greater atherogenicity [24].

To date, the most data have been collected on the importance of oxLDL [25]. The formation of oxLDL and its subsequent unregulated phagocytosis by macrophages within the arterial wall via scavenger receptors is considered to be one of the most important elements in the pathogenesis of atherosclerosis [26].

### 1.4. Oxidative Stress

Reactive oxygen species (ROS) and reactive nitrogen species (RNS), such as hydroxyl radicals, singlet oxygen, superoxide anions, hypochlorite, hydrogen peroxide, nitric oxide (NO), and peroxynitrite, are radical or non-radical compounds that arise in metabolic pathways and play an important role in physiological processes such as the control of gene expression, cell growth, transcription-factor activation, autophagy, the cell cycle, apoptosis, cell–cell interactions, intracellular signaling, and cellular defenses against pathogens [27,28]. However, under pathological conditions they can damage macromolecules essential for the function of cells, such as proteins, lipids, carbohydrates, and nucleic acids [29]. The human body has an antioxidant defense system that is responsible for protection against the excessive influence of pro-oxidative factors. Antioxidants can be divided into exogenous (such as vitamin C, vitamin E, carotenoids, and flavonoids) and endogenous types; among the endogenous antioxidants, there are enzymatic (such as superoxide dismutase, glutathione peroxidase, glutathione reductase, and catalase) and non-enzymatic (such as glutathione, ferritin, transferrin, ceruloplasmin, albumin, bilirubin, and uric acid) antioxidants [30]. The endogenous sources of ROS and RNS include the mitochondria, xanthine oxidase, lipoxygenase, myeloperoxidase (MPO), NADPH oxidase, cytochrome P450, and inducible nitric oxide synthase, among others [31,32].

The term oxidative stress (OS) refers to a greater activity of pro-oxidative factors over antioxidant factors [33]. OS has been shown to be related to the pathogenesis of various diseases such as cancer, CVDs, diabetes, obesity, neurodegenerative diseases, ankylosing spondylitis, chronic obstructive pulmonary disease, and obstructive sleep apnea [34,35,36]. Significant differences in the parameters of OS were found between obese, metabolically unhealthy patients, and healthy volunteers with normal body weight [37]. Interestingly, “obesity and insulin resistance” is the component of the metabolic syndrome most strongly associated with the presence of OS (according to principal component analysis) [38].

The possible use of nutraceuticals and supplements with antioxidant properties in the prevention and treatment of diseases remains an issue of great interest and is widely researched and discussed [39,40,41].

### 1.5. The Purpose of This Paper

The purpose of this paper is to provide an extensive review of the literature and to present the most important information on the current state of knowledge on the association of nitrotyrosine and nitrated lipoproteins with the development of the cardiovascular system dysfunction observed in patients living with DM. Moreover, directions for future research in this area are discussed.

## 2. Nitrotyrosine, Diabetes, and Cardiovascular Disease

Nitrotyrosine (NT-Tyr) is a product of tyrosine modification under the influence of peroxynitrite. Peroxynitrite is a potent prooxidative factor produced due to the interaction between superoxide anion with nitric oxide [42].

**Figure 1:**
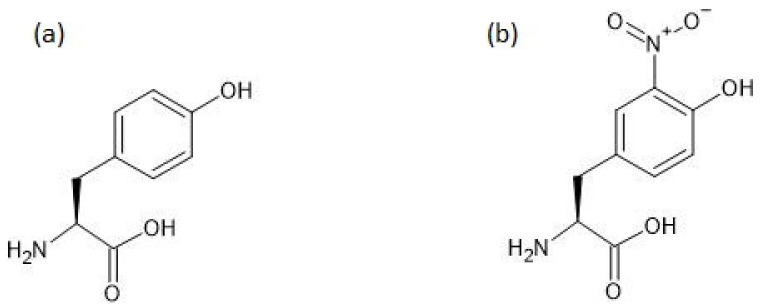
The chemical structures of tyrosine (**a**) and 3-nitrotyrosine (**b**).

The chemical structures of tyrosine and 3-nitrotyrosine are shown in Figure 1.

The NT-Tyr level was shown to be significantly higher in patients with T2DM than in non-diabetic controls, without a significant difference between patients with and without microalbuminuria among patients with T2DM [43]. On the other hand, according to Bo et al., the NT-Tyr level is significantly increased in patients with DM only in the population with a lower-than-recommended dietary intake of vitamins with antioxidant properties [44]. In a study performed by Segre et al., there was no significant difference between the level of NT-Tyr in patients with DM and angiographically confirmed CHD, and the level of NT-Tyr in patients with DM without CHD. It is worth noting that there was also no significant difference in the concentration of oxLDL, although its participation in the pathogenesis of CVD is unquestionable [45].

In vitro studies on aortic rings from non-diabetic rats have shown that canagliflozin alleviates the endothelial dysfunction induced by ischemia–reperfusion injury. The improvement in endothelial function was associated with a significant reduction in NT-Tyr content in the vessel wall. Endothelial function was assessed by the comparison of the relaxation of the smooth muscle of the aortic rings induced by an endothelium-dependent vasorelaxant (acetylcholine) and induced by an endothelium-independent vasorelaxant (sodium nitroprusside). Smooth muscles were previously contracted with phenylephrine [46].

In a study carried out on rats, it was shown that the 3-NT-Tyr level was significantly higher in an acute-blood-glucose-fluctuation group than in a constant-high-glucose group. Similarly, in the group with acute fluctuations in blood glucose, the acetylcholine-induced endothelium-dependent vascular relaxation was significantly lower than that in the group with constant high blood glucose, and the blood concentrations and expression of IL-6, TNF-α, and ICAM-1 mRNA were significantly higher. Moreover, the apoptosis index of endothelial cells was significantly higher in the group with the higher 3-NT-Tyr level [47]. It was found that supplementation with n-3 polyunsaturated fatty acids reversed endothelial dysfunction and normalized the reduction in endothelial nitric oxide synthase expression in aortas from rats with chronic kidney disease (CKD), which is associated with a substantial reduction in 3-NT-Tyr levels [48].

The Mediterranean diet was shown to significantly reduce the NT-Tyr level at three-month follow-up in patients with T2DM (0.64 ± 0.03 µmol/L at baseline vs. 0.35 ± 0.02 µmol/L at three-month follow-up; *p* < 0.05). It was associated with a significant improvement in endothelial function, as assessed by the flow-mediated dilation (FMD) method (5.6 ± 0.5% at baseline vs. 7.9 ± 0.4% at three-month follow-up; *p* < 0.05) [49]. In a study on OS and endothelial dysfunction in patients with human immunodeficiency virus (HIV) infection, it was found that NT-Tyr levels were significantly associated with carotid-radial pulse wave velocity (PWV) and that participants’ HIV status had a significant influence on this relationship [50].

Beckmann et al. demonstrated the presence of proteins whose tyrosyl residues had been nitrated in atherosclerotic lesions originating from human coronary arteries [51]. The study using immunohistochemical staining showed that the NT-Tyr content in sections taken from the renal artery and the external iliac artery was significantly higher in patients with end-stage renal disease (ESRD) eligible for transplantation than in the control group (donors of kidneys for transplantation). Moreover, among patients with ESRD, the NT-Tyr staining was significantly stronger in arteries with intima calcification, media calcification, and intima–media calcification than in arteries without the calcification of the appropriate layer of the artery wall [52]. In patients with ESRD, the expression of NT-Tyr was also shown to be significantly higher than in controls in the walls of small arteries [53]. According to Paier et al., the NT-Tyr expression in small arteries taken from subcutaneous tissue is significantly higher in patients with CHD but only in women [54]. Thus, the histopathological studies carried out to date on the sections of the artery wall suggest that, on the one hand, the amount of NT-Tyr is greater in patients with a worse cardiovascular condition, and on the other hand, in specific patients, it is greater in vascular beds with more advanced atherosclerotic processes.

The use of metformin in patients with prediabetes undergoing coronary artery bypass grafting (CABG) due to acute myocardial infarction was recently shown to be associated with a significant decrease in the concentration of NT-Tyr and proinflammatory cytokines in homogenates obtained from pericoronary fat [55]. The beneficial effect of metformin on cardiovascular risk has been documented in several meta-analyses, although it is not unambiguous [56,57,58,59].

In studies carried out in rats, it was found that drugs such as empagliflozin [60], sitagliptin [61], and spironolactone [62] showed cardioprotective potential and were able to reduce oxidative and nitrosative stress, which is associated with a reduction in NT-Tyr expression. Liraglutide has been shown to improve endothelial function in mice with polymicrobial sepsis, and to decrease the plasma concentration of 3-NT-Tyr [63].

NT-Tyr expression was shown to be significantly higher in small mesenteric arteries in mice with T2DM compared to control mice, as well as being reduced after bariatric surgery [64]. In rats with diabetic cardiomyopathy, valsartan has been shown to inhibit cardiomyocyte apoptosis by inhibiting the expression of 3-NT-Tyr [65].

### 2.1. Cellular and Molecular Mechanisms

The level of 3-NT-Tyr is a biomarker of OS and nitrosative stress that leads to cell death through mechanisms such as apoptosis, autophagy, ferroptosis, pyroptosis, NETosis, and parthanatos [66]. It was found that the nitration of protein and peptide tyrosyl residues was associated with a decrease in their ability to be degraded in proteasomes [67].

The overexpression of 3-NT-Tyr induced by a high glucose level was shown to be associated with the downregulation of peroxisome proliferator-activated receptor β (PPARβ) [68], whose activity has previously been documented to prevent endothelial dysfunction in diabetic rats [69]. This phenomenon shows a potential pathophysiological relationship between increased expression of 3-NT-Tyr and the development of CVD over the course of DM.

In a study using the rat thoracic aorta, free 3-NT-Tyr was documented to contribute to the development of endothelial dysfunction by promoting DNA damage and apoptosis [70]. NT-Tyr may also directly increase aortic smooth muscle cell migration in vitro and contribute to the overexpression of migration-related molecules through ROS production and the activation of the extracellular signal-regulated kinase 1/2 (ERK1/2) pathway [71].

Interestingly, in a human study, it was shown that the intravenous infusion of angiotensin II led to a significant increase in the concentration of NT-Tyr in the blood, and the prior inhibition of the activity of cyclooxygenase-2 (COX-2) was associated with a significantly smaller increase in the concentration of NT-Tyr [72]. It should be noted that the role of angiotensin II in the pathogenesis of CVD is well established [73] and that COX-2 plays an important role in the development of angiotensin II-induced inflammation within the vascular wall [74]. The presented relationship is, therefore, a factor supporting the role of NT-Tyr in the development of cardiovascular dysfunction, at least as a biomarker.

### 2.2. Association between Nitrotyrosine and Selected Risk Factors of CVD

The mean concentration of 3-NT-Tyr was shown to be significantly higher in smokers. Furthermore, eating foods with possibly high levels of acrylamide and drinking alcohol is associated with an increase in 3-NT-Tyr levels [75]. In an animal model study, it was shown that a diet rich in oxidized plant sterols increases the production of 3-NT-Tyr and the synthesis of cytokines (TNF-α, IL-1β, and IL-6) that can lead to a secondary disorder of lipid metabolism [76]. According to another publication, NT-Tyr expression was documented to be significantly higher in smokers than in control subjects, as well as similar in passive and active smokers [77]. On the other hand, Jin et al. documented significantly lower 3-NT-Tyr levels in the plasma proteins of smokers. It is worth noting that the methodology used in this study was used to determine the level of 3-NT-Tyr for twenty-four plasma proteins separately and not to determine the total level of protein-bound NT-Tyr. Furthermore, in the same study, it was elucidated that smokers diagnosed with chronic obstructive pulmonary disease (COPD) had significantly higher levels of NT-Tyr than smokers without COPD [78].

The NT-Tyr plasma concentration is significantly higher in sixty-year-old and older morbidly obese subjects than in morbidly obese individuals aged between twenty and thirty-nine years [79]. According to Fenster et al., weight loss (to the normal range) is associated with a significant decrease in the NT-Tyr blood level in overweight Caucasian women, but not in overweight African American women [80]. The 3-NT-Tyr levels in obese children aged three to six years are significantly higher than those in normal-weight children [81]. According to Choromańska et al., the plasma level of NT-Tyr was shown to be significantly higher in morbidly obese subjects than in lean individuals and to decrease significantly after bariatric surgery [82].

The blood levels of NT-Tyr are significantly higher in subjects with metabolic syndrome (MS) than in healthy controls (234.3 ± 158.2 µmol/mol tyrosine vs. 53.7 ± 46.8 µmol/mol tyrosine; *p* < 0.0001). Moreover, lifestyle modifications (supervised aerobic exercise and the Mediterranean diet) lead to significant decreases in NT-Tyr levels (234.3 ± 158.2 µmol/mol tyrosine vs. 58.9 ± 55.0 µmol/mol tyrosine; *p* < 0.0001) [83]. According to another study, the blood NT-Tyr levels are also significantly higher in MS subjects than in healthy controls, and a linear increase in the number of features of MS with the NT-Tyr level was observed (*p* < 0.02) [84]. In non-obese women with polycystic ovary syndrome, IR was shown to be associated with an increase in NT-Tyr levels [85]. Treatment with irbesartan (150 mg twice daily) was shown to be associated with a significant reduction in fasting NT-Tyr blood levels, as well as the levels during oral glucose tolerance tests in non-hypertensive individuals with T2DM [86].

### 2.3. Significance of Nitrotyrosine Measurements in Diagnosis of CVD and Prognosis

To date, only a small amount of research on the diagnostic and prognostic value of determining NT-Tyr concentrations in the context of CVDs has been conducted, with no consensus.

There was no significant difference in NT-Tyr blood levels between middle-aged men with arterial hypertension, middle-aged men with arterial hypertension and other cardiovascular disorders, and a control group [87]. According to Shishehbor et al., the NT-Tyr levels were significantly higher in patients with CHD than in control subjects (9.1 µmol/mol vs. 5.2 µmol/mol, respectively; *p* < 0.001). It should be noted that the correlation remained significant after adjustment for the Framingham Global Risk Score, age, sex, DM, arterial hypertension, current smoking, HDL-C, LDL-C, TG, and CRP. Moreover, statin therapy was shown to significantly reduce NT-Tyr levels (25%; *p* < 0.02) [88]. Four-week rosuvastatin therapy (10 mg daily) was shown to be associated with a significant reduction in NT-Tyr blood concentrations [89]. According to Ferlazzo et al., the plasma 3-NT-Tyr level was significantly higher in patients with periodontitis and CHD in comparison to healthy subjects, although there was no significant difference between patients with periodontitis and healthy subjects, or between patients with CHD and healthy subjects [90].

The NT-Tyr concentration was shown to have no prognostic value in terms of survival after acute coronary syndrome at a four-year follow-up [91]. In a study conducted by Heslop et al., it was shown that there were no significant differences in the concentrations of NT-Tyr in the blood between patients with confirmed or excluded features of CHD using angiography (72.1 vs. 71.9 nmol/L, respectively; *p* = 0.965). In the same study, the risk of cardiovascular mortality increased across tertiles of blood concentrations of NT-Tyr. The difference between the highest and lowest tertiles became statistically significant after four years of follow-up. However, after multivariate adjustment for factors such as age, sex, TC/HDL-C ratio, body mass index, smoking, DM, and hypertension, attenuation of the correlation was observed (*p* = 0.08), which indicates that the NT-Tyr level is not an independent predictor of cardiovascular mortality [92].

The NT-Tyr blood level was suggested to be a marker of transient cardiac ischemia following coronary vasospasm. In patients with vasospastic angina pectoris (VSAP), the blood levels of NT-Tyr increased significantly three and twelve hours after an acetylcholine provocation test, with no significant differences fifteen minutes after the test, while in the control group, the serum level of NT-Tyr decreased significantly [93]. In another study, no significant differences in the levels of NT-Tyr thirty minutes after an exercise test in patients with positive exercise tests, or in patients with negative exercise tests, were observed [94].

In 2021, a paper was published in which a real-time 3-NT-Tyr detection system using a smartphone-based electrochemiluminescence system was presented [95]. Undoubtedly, it is an interesting invention, but its usefulness in clinical practice requires further research.

## 3. Nitrated Lipoproteins and Diabetes Mellitus

Postprandial hyperglycemia elevates the risk of ROS overproduction, as well as increasing the synthesis of non-enzymatic early glycated and nitrated proteins, and it makes the lipoprotein profile more atherogenic [96]. Lipoproteins can undergo MPO-catalyzed enzymatic nitration, and the reaction concerns the apolipoprotein apoA-I in HDL particles and apoB in LDL particles [97].

Alterations in MPO activity have been investigated over the course of DM. According to Peng et al., the concentrations of serum extracellular-vesicle-derived MPO as well as serum MPO were shown to be significantly higher in patients with T2DM than in controls without DM [98]. A similar conclusion related to an increase in MPO levels in DM patients was previously documented in other publications [99,100,101]. On the other hand, papers reporting no significant differences in MPO levels between patients with and without DM have been published [102,103]. Thus, the results of studies on the relationship between DM and MPO levels are not conclusive and further research is needed.

The blood levels of nitrated apolipoprotein A-I (NT-apoA-I) were significantly higher in patients living with T2DM than in age-matched control subjects [104]. In patients with T2DM, an increased blood level of NT-apoA-I is associated with a decrease in serum paraoxonase-1 (PON-1) activity [105]. PON-1 is responsible for the antioxidant properties of HDL particles [106]. DM was shown to be an independent predictor of a higher NT-apoA-I/apoA-I ratio, and this value is negatively correlated with cholesterol efflux from macrophages independently of total apoA-I levels [107].

According to Lu et al., MPO bound to apoA-I in serum taken from patients with T2DM facilitated the selective oxidative modification of HDL particles. Furthermore, using liquid chromatography with tandem mass spectrometry, Tyr192 was shown to be the main nitration and chlorination site in the apoA-I present in serum taken from patients living with T2DM. It was also documented that the apoA-I nitration and chlorination levels increase in patients with T2DM. The oxidative modification of apoA-I mediated by MPO is associated with a decrease in the cholesterol efflux properties and antiapoptotic activity (through diminished possibilities for the inhibition of caspase-3 activity) of HDL particles [108].

## 4. Nitrated Lipoproteins and Cardiovascular Disease

According to Shao et al., Tyr192 is the major nitration site in the apoA-I of circulating HDL particles, while the highest nitration level in the apoA-I in HDL particles within atherosclerotic lesions is suspected to be associated with Tyr18, although without a significant difference from that for Tyr100 [109]. In atheromatous plaques taken from human aortas, apoA-I particles were shown to have undergone MPO-dependent nitration at residues Tyr192 and Tyr166 [110]. Apolipoprotein A-I nitrated at Tyr166 accounts for 8% of the total apoA-I in atherosclerotic coronary arteries, but over 100-fold less in normal coronary arteries [111].

The MPO-dependent oxidative modification of HDL particles leads to a change in cholesterol transport through the ATP-binding cassette transporter A1 (ABCA1) pathway [112]. LDL nitration is associated with the formation of cholesterol-rich cells, a key feature of atherosclerosis [113].

**Figure 2:**
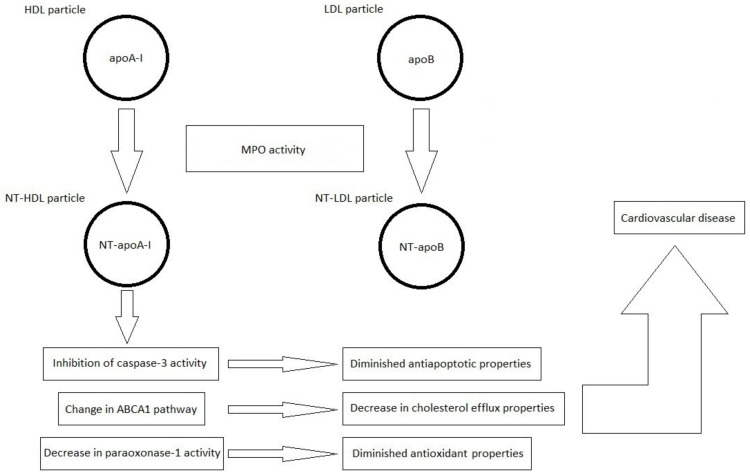
Formation of nitrated lipoproteins (NT-HDL and NT-LDL) under the influence of myeloperoxidase (MPO) activity. Nitrated lipoproteins are characterized by the presence of 3-NT-Tyr in the polypeptide chains of apolipoprotein A-I (apoA-I) and apolipoprotein B (apoB), respectively, resulting in the formation of nitrated apoA-I (NT-apoA-I) and nitrated apoB (NT-apoB), respectively.

The most important information in the field of current knowledge on the formation of nitrated lipoproteins and their importance in the pathogenesis of CVDs is schematically summarized in Figure 2.

The blood concentration of nitrated high-density lipoproteins (NT-HDL) was shown to be a useful early marker of microangiopathic dysfunction, as assessed using the vascular reactivity index (VRI), in African American patients with T2DM, in the total population, and in subjects with HbA1c ≤ 7.0%. In the same study, the blood NT-LDL concentration was shown to be significantly associated with the carotid intima–media thickness (CIMT) outcome in the total population and in patients with HbA1c > 7% but only in a univariable analysis. In a multivariate analysis, the significant association was lost. Sex and the duration of DM were confounding variables that affected the association between NT-LDL and CIMT [114].

## 5. Factors Influencing Blood Concentration of Nitrated Lipoproteins

According to Mathew et al., a twelve-week lifestyle modification (exercise and the Mediterranean diet) led to a significant decrease in the 3-NT-Tyr levels in HDL particles in subjects diagnosed with metabolic syndrome. Similarly, a significant decrease in the level of 3-chlorotyrosine was observed in HDLs. This is suspected to be the direct effect of the decrease in MPO activity [115].

To our knowledge, Mathew et al.’s study is currently the only one available in the literature that has shown the effect of lifestyle modifications on the concentration of nitrated lipoproteins. During the preparation of this paper, we did not find any study in which the influence of pharmacological treatment on the concentration of nitrated lipoproteins was examined. However, there are data that indicate a beneficial effect of lifestyle modifications and drugs used in CVD treatment on the activity of MPO, which is considered an important source of NT-Tyr within lipoprotein molecules.

Twelve-week treatment with atorvastatin (20–40 mg daily) significantly reduced the MPO activity in the blood in patients with moderate to very high risks of atherosclerotic CVD [116]. In patients with acute coronary syndrome, who received rosuvastatin on the first day, a significantly greater decrease in MPO activity was observed compared to that in the placebo group [117]. In patients admitted to hospital for coronary angiography, the use of drugs such as beta-blockers, statins, and angiotensin-converting-enzyme inhibitors was observed to be associated with significantly lower levels of MPO blood activity in patients with acute coronary syndrome, but not in patients with stable coronary syndrome. In multivariate analysis, the effect remained statistically significant for beta-blockers [118].

NT-apoA-I was documented to be significantly reduced at twelve months after kidney transplantation, as well as associated with a significant reduction in MPO activity. On the other hand, nitrated apolipoprotein B (NT-apoB) did not change significantly after kidney transplantation [97]. In a recently published epidemiological study, it was shown that, in a fifteen-year observation, the risk of death over the course of CVD was lower in the population of patients with CKD after transplantation than in patients who did not undergo transplantation (2.3% vs. 15.2%) [119]. This suggests that NT-apoA-I may become a new marker for the evaluation of cardiovascular risk in patients with CKD in the future, but its usefulness in clinical practice needs to be further evaluated.

## 6. Conclusions and Directions for Future Research

The results of this review of the literature show that the level of NT-Tyr is a widely used marker in research, and the results provide premises indicating a significant relationship between NT-Tyr and CVD. The most important findings of our review of the literature related to NT-Tyr are presented in Table 2.

Based on the studies conducted to date, the relationship between increased levels of NT-Tyr and dysfunction of the cardiovascular system seems to be unquestionable, although the data on the usefulness of this parameter in the assessment of prognosis in patients with CVD are more ambiguous.

The importance of peroxynitrite, under the influence of which NT-Tyr is formed, in the context of cardiovascular dysfunction in DM was the subject of a review published by Pacher and Szabó in 2006. The authors of this study drew similar conclusions indicating that NT-Tyr is, on the one hand, a marker of dysfunction of the cardiovascular system during DM. On the other hand, it also probably plays a direct pathogenetic role. We had more data in the preparation of this publication, as we, of course, also used papers published after 2006, which makes this review more up to date. In this paper, we are also concerned with the issue of nitrated lipoproteins, which was not discussed in the publication mentioned above [42].

The term nitrated lipoproteins refers to those lipoprotein molecules in which the tyrosyl residues are nitrated in the polypeptide chain of the apolipoprotein. Our review of the literature shows that, contrary to NT-Tyr research, little research on nitrated lipoproteins and their role in the pathogenesis of CVD has been conducted to date, the information on which remains limited. The limitation of this literature review is the relatively small number of publications on nitrated lipoproteins. The most important findings of our review of the literature related to nitrated lipoproteins and their role in the pathogenesis of CVDs are summarized in Table 3.

Undoubtedly, OS plays an important role in the pathogenesis of atherosclerotic CVD. An increased level of NT-Tyr is one of the markers of OS. It is difficult to say whether the increased level of nitrated lipoproteins is only a marker of OS that provides no additional information with the level of NT-Tyr, or whether it provides additional information and plays an independent role in the pathogenesis of atherosclerotic CVD. More detailed research is needed to dispel these doubts. The study of the importance of nitrated lipoproteins in the development of cardiovascular dysfunctions in patients with DM is an interesting direction for further research. In our opinion, the issue of the role of nitrated lipoproteins in the development of cardiovascular dysfunction could be the subject of a large clinical research project involving patients living with DM and controls. Table 4 presents the research questions that should be addressed. We hope that this may also inspire other research teams that wish to conduct research on such topics. More work is also required at the level of basic research to better understand how nitrated lipoproteins are formed, how nitration alters lipoprotein function, and how it can contribute to the development of CVD, including in patients with DM.

## Figures and Tables

**Table 1:** Results of selected studies on the relationship between insulin and lipid metabolism.

In a study on rats, it was shown that hyperinsulinemia induced by intravenous glucose administration inhibited the hepatic secretion of VLDL molecules (the reduction in TG secretion was 30% and the reduction in apolipoprotein B (apoB) secretion was 66%) [14].
In a study performed on hepatocytes derived from mice, the suppressive effect of insulin on VLDL secretion was not dependent on LDL-receptor function [15].
In Zucker diabetic fatty rats, increased TG production over the course of hyperinsulinemia was shown to be related to the increased expression of sterol regulatory element-binding protein 1c (SREBP-1c), whereas increased apoB production involved posttranscriptional processes [16].
In an IR model of fructose-fed hamsters, the activity of phosphatidylinositol 3-kinase was shown to be significantly reduced, as well as the activity of protein-tyrosine phosphatase-1B (PTP-1B) being significantly higher, in the hepatocytes of these animals. Interestingly, the increase in PTP-1B was associated with the marked suppression of a cysteine protease (ER-60) playing a role in intracellular apoB degradation [17].
The activity of microsomal triglyceride transfer protein (MTP) was shown to be significantly increased in an animal model of T2DM [18] and in studies performed on hepatocytes [19,20]. The MAPK pathway was shown to participate in the insulin-mediated inhibition of MTP synthesis [20]. Moreover, increased transcription factor forkhead box O1 (FoxO1) and decreased transcription factor forkhead box A2 (Foxa2) activity may participate in the regulation of VLDL excretion mediated by insulin [21,22].

**Table 2:** Associations between 3-nitrotyrosine and cardiovascular disease—the most important findings.

3-nitrotyrosine overexpression has been shown to be associated with endothelial dysfunction [46,47,48,49,50].
The 3-nitrotyrosine content in the arterial wall is higher in patients with worse cardiovascular system conditions [51,52,53,54].
Certain drugs used in the treatment of diabetes mellitus and CVDs can reduce the expression of 3-nitrotyrosine [55,60,61,62,63,65].
Most of the studies cited indicate that the expression of 3-nitrotyrosine generally tends to increase with the presence of cardiovascular risk factors such as age, obesity, smoking, consumption of highly processed foods, and the presence of features of metabolic syndrome [75,76,77,78,79,80,81,82,83,84,85,86].
There are currently no unambiguous data that would allow the use of the determination of the level of 3-nitrotyrosine in the diagnosis of CVDs and in the assessment of prognosis [87,88,89,90,91,92,93,94].

**Table 3:** Nitrated lipoproteins, diabetes mellitus, and cardiovascular disease—the most important findings.

The nitrated lipoproteins NT-HDL and NT-LDL result from the nitration of tyrosyl residues in the polypeptide chain of apoA-I and apoB, respectively [97].
Lipoprotein nitration is influenced by the catalytic activity of myeloperoxidase, which is suspected to be increased in patients with DM, although this is not unambiguous [98,99,100,101,102,103].
Currently, more information on nitrated HDL particles than on nitrated LDL particles is available in the literature.
The nitration of HDL molecules is associated with a decreased activity of paraoxonase-1 and caspase-3 [105,108], and also influences the transport of cholesterol via ABCA1 [112].
The mechanisms mentioned above are associated with a reduction in the antioxidant and antiapoptotic properties of HDL particles and a reduction in the ability to transport cholesterol. Therefore, nitrated HDL particles have weaker antiatherogenic properties than native HDL particles [105,108,112].

**Table 4:** The role of nitrated lipoproteins in the pathogenesis of cardiovascular disease—perspectives for future research.

Research Question
Is there a relationship between the concentration of nitrated lipoproteins and parameters of glycemic control in patients with diabetes?
Is there a relationship between the concentration of nitrated lipoproteins and the systemic parameters of oxidative stress in patients with diabetes?
Is there a relationship between the concentration of nitrated lipoproteins and the characteristics of myocardial systolic and/or diastolic dysfunction in patients with diabetes?
Is there a relationship between the concentration of nitrated lipoproteins and the characteristics of subclinical dysfunction of the cardiovascular system measured with parameters such as flow-mediated dilation, intima–media thickness, pulse-wave velocity, ankle-brachial index, and toe-brachial index in patients with diabetes?
Is there a relationship between the concentration of nitrated lipoproteins and anthropometric parameters (body mass index, waist circumference, waist–hip ratio, and body composition analysis results) in patients with diabetes?
Can the concentration of nitrated lipoproteins be a useful marker of the risk of developing CVD and the risk of cardiovascular events in a prospective observation, and is the concentration of nitrated lipoproteins in this range a parameter independent of the concentration of nitrotyrosine and myeloperoxidase activity?

## Abbreviations

ABCA1	ATP-binding cassette transporter A1
apoA-I	apolipoprotein A-I
apoB	apolipoprotein B
CABG	coronary artery bypass grafting
CETP	cholesteryl ester transfer protein
CHD	coronary heart disease
CIMT	carotid intima–media thickness
CKD	chronic kidney disease
COPD	chronic obstructive pulmonary disease
COX-2	cyclooxygenase-2
CVD	cardiovascular disease
DM	diabetes mellitus
ESRD	end-stage renal disease
FFA	free fatty acid
FMD	flow-mediated dilation
FoxO1	transcription factor forkhead box O1
Foxa2	transcription factor forkhead box A2
HDL	high-density lipoprotein
HDL-C	high-density lipoprotein cholesterol
HIV	human immunodeficiency virus
ICVD	ischemic cerebrovascular disease
IR	insulin resistance
LDL	low-density lipoprotein
LDL-C	low-density lipoprotein cholesterol
MPO	myeloperoxidase
MS	metabolic syndrome
MTP	microsomal triglyceride transfer protein
NO	nitric oxide
NT-apoA–I	nitrated apolipoprotein A–I
NT-apoB	nitrated apolipoprotein B
NT-HDL	nitrated high-density lipoprotein
NT-LDL	nitrated low-density lipoprotein
NT-Tyr	nitrotyrosine
OS	oxidative stress
oxLDL	oxidized LDL particle
PAD	peripheral arterial disease
PON-1	paraoxonase-1
PPARβ	peroxisome proliferator-activated receptor β
PTP-1B	protein-tyrosine phosphatase-1B
PWV	pulse-wave velocity
ROS	reactive oxygen species
RNS	reactive nitrogen species
sdLDL	small dense low-density lipoprotein
SREBP1c	sterol regulatory element-binding protein 1c
TG	triglycerides
T1DM	type 1 diabetes mellitus
T2DM	type 2 diabetes mellitus
VLDL	very low-density lipoprotein
VRI	vascular reactivity index
VSAP	vasospastic angina pectoris
